# Differential Neural Responses Underlying the Inhibition of the Startle Response by Pre-Pulses or Gaps in Mice

**DOI:** 10.3389/fncel.2017.00019

**Published:** 2017-02-07

**Authors:** Rocio Moreno-Paublete, Barbara Canlon, Christopher R. Cederroth

**Affiliations:** Laboratory of Experimental Audiology, Department of Physiology and Pharmacology, Karolinska InstitutetStockholm, Sweden

**Keywords:** tinnitus, pre-pulse inhibition, gap detection, auditory cortex, LGP, mouse models, dopamine, hearing loss, c-fos, neural mapping, catecholamines, NMDA receptor

## Abstract

Gap pre-pulse inhibition of the acoustic startle (GPIAS) is a behavioral paradigm used for inferring the presence of tinnitus in animal models as well as humans. In contrast to pre-pulse inhibition (PPI), the neural circuitry controlling GPIAS is poorly understood. To increase our knowledge on GPIAS, a comparative study with PPI was performed in mice combining these behavioral tests and c-Fos activity mapping in brain areas involved in the inhibition of the acoustic startle reflex (ASR). Both pre-pulses and gaps efficiently inhibited the ASR and abolished the induction of c-Fos in the pontine reticular nucleus. Differential c-Fos activation was found between PPI and GPIAS in the forebrain whereby PPI activated the lateral globus pallidus and GPIAS activated the primary auditory cortex. Thus, different neural maps are regulating the inhibition of the startle response by pre-pulses or gaps. To further investigate this differential response to PPI and GPIAS, we pharmacologically disrupted PPI and GPIAS with D-amphetamine or Dizocilpine (MK-801) to target dopamine eﬄux and to block NMDA receptors, respectively. Both D-amp and MK-801 efficiently decreased PPI and GPIAS. We administered Baclofen, an agonist GABA_B_ receptor, but failed to detect any robust rescue of the effects of D-amp and MK-801 suggesting that PPI and GPIAS are GABA_B_-independent. These novel findings demonstrate that the inhibition of the ASR by pre-pulses or gaps is orchestrated by different neural pathways.

## Introduction

Pre-pulse inhibition (PPI) is a quantitative measure of the sensorimotor gating where a pre-pulse attenuates the motor reflex that is induced by a subsequent acoustic startle. The acoustic startle reflex (ASR) is a primitive survival reaction relying on the dorsal cochlear nucleus (DCN), the caudal pontine reticular nucleus (PnC), and spinal motor neurons (Koch, 1999). Higher order nuclei including the limbic system and the prefrontal cortex regulate the inhibition of the ASR during PPI. It is known that the activation of dopamine and the blockade of NMDA receptors can disrupt the function of the pre-frontal cortex and the nucleus accumbens and lead to impaired PPI (Koch and Schnitzler, 1997). Altered PPI responses are found in a variety of psychiatric disorders including schizophrenia, obsessive–compulsive disorder, and Tourette’s syndrome (Braff et al., 2001; Swerdlow et al., 2008, 2016). Enhancing GABAergic inhibition has been used as strategy to circumvent the disrupted dopamine/glutamate circuitry and to restore the inhibitory inputs to the PnC and PPI (Braff et al., 2001; Swerdlow et al., 2008, 2016).

A variant to PPI is gap pre-pulse inhibition of the acoustic startle response (GPIAS), which has emerged as a potential tool for the assessment of tinnitus in animals and humans (Galazyuk and Hebert, 2015). Conceived by Turner et al. (2006) and Turner and Parrish (2008) it was validated against a model of operant conditioning and was further supported by additional neuronal correlates of tinnitus including increased spontaneous firing rates (SFRs) in the DCN (Li et al., 2013, 2015), hyperactivity in the inferior colliculus (IC) (Holt et al., 2010) and remapping of the auditory cortex (AC) (Engineer et al., 2011). Unlike PPI, GPIAS uses a silent gap embedded in a carrier noise as a pre-stimulus to decrease a subsequent ASR. When the carrier frequency closely matches that of the tinnitus, it interferes with the optimal inhibition caused by the silent gap. Lowe and Walton (2015) adapted the GPIAS to assess neuronal responses instead of startle responses using a paradigm named auditory brainstem response gap-in-noise (ABR GIN), which showed similar efficacy in detecting tinnitus. How GPIAS and PPI, which are elicited by different auditory cues, differ in terms of neural mapping and pharmacological regulation, remains to be determined.

Some of the temporal characteristics and neural circuitry used to elicit inhibition with gaps or pre-pulses differ. For instance, PPI is stable within a large range of inter-stimulus intervals (ISI), whereas GPIAS improves with shorter lead times in mice depending on the strain (Yu et al., 2016). Moreover, the AC appears as an important regulator of GPIAS, but not PPI, as shown by surgical ablation studies (Bowen et al., 2003). Recent optogenetic studies in mice have shown that GABAergic interneuron activity in the AC is important in controlling perceptual gap detection (Weible et al., 2014). With the exception of the AC, little is known about the neural structures controlling GPIAS. In this study, we performed behavioral tests and histological evaluations based on c-Fos induction to compare GPIAS and PPI responses in the mouse.

## Materials and Methods

### Experimental Animals

Experimental procedures on animals were performed in accordance with the guidelines and regulations set out by *Stockholm’s Norra Djurförsöksetiska Nämnd* (N156/14). Male mice from 3 to 4 months of age in a C57BL/6JRj background (Janvier Labs, France), were group-housed (4–5 per cage) and maintained at 19–21°C in a 50–50% light-dark cycle (lights on at 06:00). Animals had free access water and food (Lactamin R34, Lantmännen) and were given a minimum of 1 week acclimatization upon their arrival prior to any manipulation. All behavioral experiments were carried out between 09:00 and 16:00 h.

### Gap-Pre-pulse Inhibition of the Startle Eesponse (GPIAS) and Pre-pulse Inhibition (PPI)

A SR-Lab startle response system from San Diego Instruments was used. A background carrier sound level consisted of an unfiltered white noise at 65 dB sound pressure level (SPL) for PPI. Pre-pulses of 3, 6, and 12 dB SPL above the carrier noise were used. For GPIAS, the gaps were 6, 11 and 16 dB SPL below the carrier noise, and the lowest level of the gaps was 65 dB SPL (noise floor). Both pre-pulses and gaps were 50 ms in duration and had 0.1-ms rise and fall times. Startle pulses of 20 ms were presented at 114 dB SPL. Calibration was performed before each procedure and SPL were measured with a calibrated microphone and preamplifier (4939-A-011 and 2633, Brüel & Kjær), connected to an amplifier (Brüel & Kjær, type 2636). The ISI was set at 70 ms for pre-pulses and 15 ms for silent gaps according to Yu et al. (2016). Gap detection or pre-pulse inhibition was quantified as [(1 – (startle amplitude during pre-pulse or gap + pulse)/(startle amplitude during pulse alone)) × 100)] (Yu et al., 2016), using a similar representation as used in pre-pulse inhibition studies (greater suppression of the startle reflex closer to 100%).

#### Experimental Procedure for PPI and GPIAS

A 10 min acclimatization to the procedure was conducted on day 1. On the following day (day 2), a baseline experiment comprised a complete test with both PPI and GPIAS sessions, and the same scheme was used on day 5 for the evaluation of drug effects. PPI sessions were performed first, immediately followed by GPIAS sessions. A pilot test showed that inverting PPI and GPIAS did not alter the outcome of the study in presence or absence of drugs. The PPI and GPIAS sessions were initiated after a 5 min acclimatization to the environment, followed by 5 min in a 65 dB SPL continuous white noise.

##### PPI session

The PPI session started with five startle pulses; each pulse was 20 ms in duration and at 114 dB SPL. PPI was tested at a single carrier intensity (white noise) of 65 dB SPL with trials containing pre-pulses of 3, 6, and 12 dB SPL above the carrier level. Each pre-pulse intensity was tested pseudo-randomly 10 times with eight no stimulus trials randomly inserted. The time between each trial was random and between 9 and 15 s. The session ended with five trials containing only startle pulses to be compared with those of the beginning of the session to assess habituation.

##### GPIAS session

The GPIAS session started with five startle pulses 20 ms in duration and at 114 dB SPL. GPIAS was then tested at a different carrier intensities (white noise) with trials containing gaps of 6, 11, and 16 dB SPL below the carrier level and reaching a floor of 65 dB SPL. Each intensity was tested pseudo-randomly 10 times with eight no stimulus trials randomly inserted. The session ended with five trials containing only startle pulses to be compared with those of the beginning to assess habituation.

**FIGURE 1:**
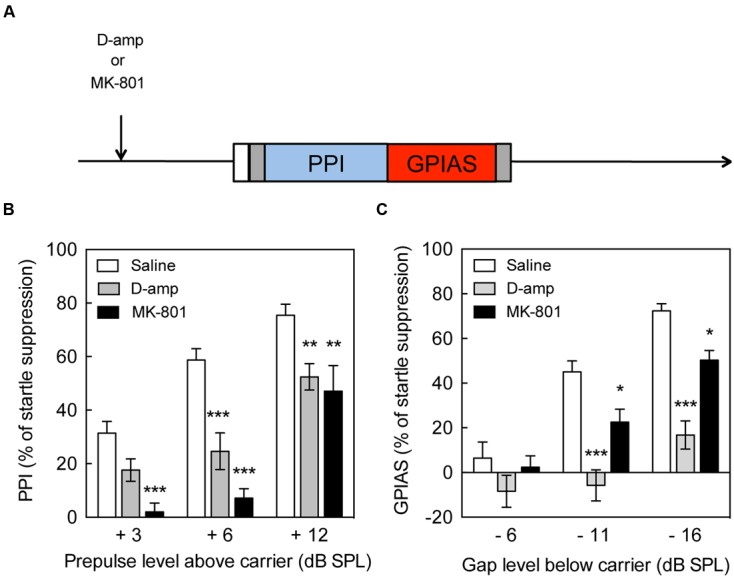
**Impairment of pre-pulse inhibition and gap pre-pulse inhibition of the acoustic startle by D-amp and MK-801. (A)** Schematic diagram of the behavioral tests performed to evaluate the effects of drugs on pre-pulse inhibition (PPI) and gap pre-pulse inhibition of the acoustic startle (GPIAS). Effect of acute D-amp (10 mg/kg, gray) and MK-801 (0.5 mg/kg, dark) administration on PPI **(B)** and GPIAS **(C)**. Data represent mean ± SEM (*n* = 13–19). ^∗^*P* < 0.05, ^∗∗^*P* < 0.01, ^∗∗∗^*P* < 0.0001.

A total of 118 trials were presented in approximately 40 min. A schematic diagram illustrating the sequence of the sessions performed on day 5 is presented in **Figure 1A**. The sequence of PPI and GPIAS trials for these sessions are provided in the supplementary method (Supplementary Data Sheet 1).

#### PPI and GPIAS Sessions for c-FOS Evaluation in the Brain

**FIGURE 2:**
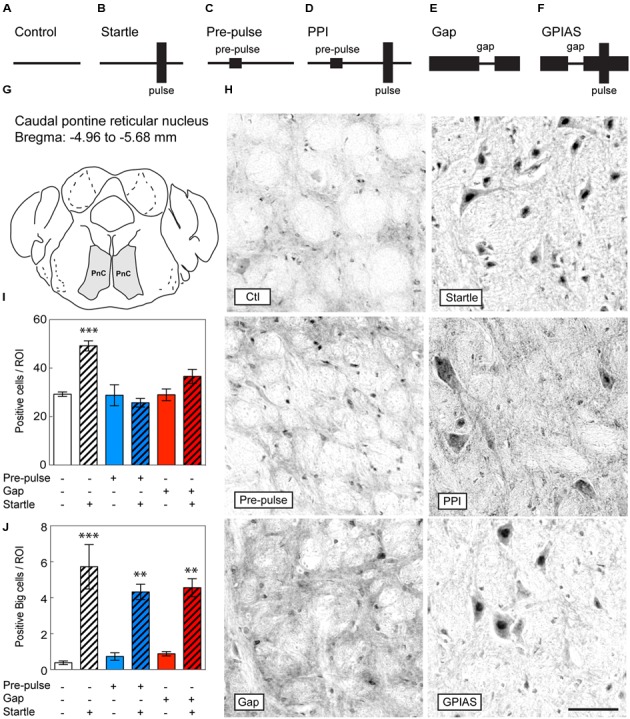
**Lack of c-Fos induction in the PnC upon inhibition by pre-pulses or gaps.** Schematic diagram of the behavioral tests performed to evaluate c-Fos induction in the brain under **(A)** only carrier noise (control), **(B)** pulses only (startle), **(C)** pre-pulses only (pre-pulse), **(D)** pre-pulses in presence of startle stimuli (PPI), **(E)** gaps only (gap), and **(F)** gaps in presence of startle stimuli (GPIAS). Sections were taken from the PnC **(G)**, which is located caudally from the bregma along the rostrocaudal axis between -4.96 and -5.68 mm. **(H)** Representative photomicrographs of c-Fos-immunostaining in the PnC under the six conditions described in **(A–F)** Scale bar, 50 μm. Quantification of c-Fos positive cells **(I)** and big neurons **(J)** in the PnC of the six groups. Groups with startle pulses are in hatched bars. Those with pre-pulses are in blue and those with gaps are in red. Data represent means ± SEM (*n* = 4). ^∗∗^*P* < 0.01, ^∗∗∗^*P* < 0.0001.

For the evaluation of c-Fos induction in the brain, mice were acclimatized to the procedure 10 min per day, for 3 consecutive days. On day 4, mice were randomly allocated to six test groups, which lasted approximately 20 min. Each session started with a 10 min acclimatization (5 min silence and 5 min background noise) followed by one session of 60 trials with a random inter-trial time interval from 10 to 20 s. The six sessions were as follows: (i) carrier noise only (65 dB SPL), (ii) startle only (60 startle pulses), (iii) pre-pulses (60 pre-pulses), (iv) PPI (60 pre-pulses followed by startle pulses), (v) gaps (60 gaps), and (vi) GPIAS groups (60 gaps followed by startle pulses). Pre-pulses used here were +12 dB SPL above carrier noise (65 dB SPL), gaps were -16 dB SPL below carrier noise (81 dB SPL), startle pulses were 20 ms in duration and 114 dB SPL. A schematic of the trials presented for the evaluation of c-Fos is shown in **Figure 2A**. The sequence of PPI and GPIAS trials used for the evaluation of c-Fos induction are provided in the supplementary method (Supplementary Data Sheet 2).

### Immunohistochemistry

Animals were anesthetized 2 h after the end of the behavioral procedure with a mixture of ketamine/xylazine (100/10 mg/kg) and underwent transcardiac perfusion with phosphate-buffered saline (PBS) and then with 4% paraformaldehyde. A pilot study found a 2 h time point to be the most effective in revealing c-Fos induction when compared to 1 or 3 h after the end of the experimental procedure. Brains were post-fixed (1 h) and then cryoprotected in 30% sucrose in PBS, mounted in NEG 50 (#6502, Thermo Scientific) and frozen prior to serial sectioning at 14 μm thickness. Sections from four animals per conditions were immunostained with rabbit-antibodies to c-Fos (#4384; 1:250; Cell Signaling technology) then incubated with biotinylated goat anti-rabbit antibodies (BA-1000; Vector Laboratories, Burlingame, CA, USA) and the avidin/biotin system (SP-2001; Vector Laboratories, Burlingame, CA, USA) and visualized using 3, 3′-diaminobenzidine (DAB) solution (SK-4100; Vector Laboratories). Negative controls were performed by omitting the primary antibody yielding no staining. Images were obtained using DP Controller software (Olympus, Tokyo, Japan) and immunopositive nuclei were counted automatically using Image-Pro Plus 6.2 software (Media Cybernetics, Rockville, MD, USA). Pilot studies were performed using counterstaining with hematoxylin QS (H-3404, Vector Laboratories) to confirm the different brain regions.

### Quantitative Analysis of c-Fos Immunohistochemistry

Serial coronal sections were examined at 10× magnification using a Zeiss Axioskop (Zeiss, Germany). Photographs were taken with an Olympus DP71 digital camera (resolution of 4140 pixels × 3086 pixels). Franklin and Paxinos (2008) stereotaxic coordinates were used to define specific brain regions: (i) the caudal pontine reticular nucleus (PnC), between -4.96 and -5.70 mm; (ii) the lateral globus pallidus (LGP), -0.10 and -1.06 mm; and the auditory cortex (AC), in the region between -2.18 and -3.64 mm. Both right and left hemispheres of three different sections separated by at least 140 μm for each area were examined. Within each section 3 regions of interest (ROI) were analyzed. The size of the three ROI was 200 × 200 μm. Using Image-pro 6.2.1 (Media Cybernetics) and ImageJ 1.50i (NIH), cells with threshold above background were counted. The researcher performing the analysis was blind to the experimental conditions. This procedure resulted in a total of 18 determinations of the number of cells stained with c-Fos within a specified area for each brain.

### Pharmacological Procedures

For the pharmacological evaluation of drugs on PPI and GPIAS, animals were acclimatized to the procedure on day 1, baseline levels were tested on day 2 and drug treatment effects were tested on day 5. Mice were injected subcutaneously (s.c.) with 7.5 mg/kg (±) baclofen (B5399; Sigma–Aldrich), followed 15 min after with 10 mg/kg D-amphetamine hemisulfate salt (A5880, Sigma–Aldrich) or 0.5 mg/kg MK-801 (M107; Sigma–Aldrich). For the use of MK-801, we initially tested a 1 mg/kg dose as previously reported in the literature (Arai et al., 2008), however, mice appeared lethargic and therefore used a 0.5 mg/kg dose, which was tolerated better (qualitative observations). Drugs were dissolved in physiological saline and administered at a volume of 0.2 ml/30 g body weight. The PPI/GAP test was performed 30 or 15 min after the last administration of D-Amp or MK-801, respectively (**Figure 1A**). Saline was used as vehicle and control treatment.

### Statistical Analyses

One-way ANOVA and a Tukey *post hoc* test were used for all histological quantifications, and a two-way ANOVA and a Bonferroni *post hoc* test were used in the context of GPIAS and PPI (Prism version 4.0, GraphPad software). Differences were considered significant if *p* < 0.05. Animals that failed to respond to the startle (any peak-to-peak response above noise floor) or failed to inhibit the startle in the presence of a pre-pulse before treatment (any decrease in startle amplitude during pre-pulse trials versus startle only trials) were excluded from the analysis (near 5%).

## Results

### Disruption of PPI and GAP by D-Amp and MK-801

The paradigm used for the drug treatment is illustrated (**Figure 1A**) and shows the sequence of drug administration and behavioral tests. PPI increased up to 75% suppression of the startle response in presence of a +12 dB pre-pulse, whereas GPIAS achieved near 72% suppression with a -16 dB gap. These results are illustrating that the paradigm between the two tests is relatively equal, and a progressive increase in inhibition with increasing pre-pulse or carrier levels was achieved. D-amp suppressed PPI by 23–36% in the +6 and +12 dB SPL pre-pulse intensities [Treatment Factor, *F*(1,102) = 36.35, *p* < 0.0001; Carrier Intensity Factor, *F*(2,102) = 30.83, *p* < 0.0001; **Figure 1B**]. Similarly, D-amp suppressed GPIAS by 49–54% in the -11 and -16 dB SPL gaps [Treatment Factor, *F*(1,96) = 54.54, *p* < 0.0001; Carrier Intensity Factor, *F*(2,96) = 25.43, *p* < 0.0001; **Figure 1C**]. MK-801 suppressed PPI by 53% in the +6 and +12 dB SPL pre-pulse intensities [Treatment Factor, *F*(1,87) = 75.95, *p* < 0.0001; Carrier Intensity Factor, *F*(2,87) = 38.75, *p* < 0.0001; **Figure 1B**]. Similarly, MK-801 suppressed GPIAS by 41–50% in the -11 and -16 dB SPL gaps [Treatment Factor, *F*(1,96) = 54.54, *p* < 0.0001; Carrier Intensity Factor, *F*(2,96) = 25.43, *p* < 0.0001; **Figure 1C**]. These findings show that -16 dB SPL gaps inhibit the startle response to a level equivalent to a +12 dB SPL pre-pulses, and that both PPI and GPIAS are disrupted by the rise in available dopamine and NMDA receptor antagonism. Moreover, the data suggest that GPIAS is more vulnerable to D-Amp than PPI and this was confirmed by measuring the change caused by D-Amp versus the individual’s baseline values [*q*(180) = 4.453, *p* = 0.0103, data not shown].

### PPI and GPIAS Elicit Similar c-FOS Responses in the PnC

To identify the differential neural circuits underlying PPI and GPIAS, we evaluated c-Fos induction in the brain by immunohistochemistry from mice under the following conditions: (i) control (carrier noise only), (ii) startle only, (iii) pre-pulses only, (iv) PPI (pre-pulses in presence of startle stimuli), (v) gaps only, and (vi) GPIAS (gaps in presence of startle stimuli) (**Figures 2A–F**). We hypothesized that regions that specifically regulating PPI and GPIAS would show a differential induction of c-Fos. When first assessing the PnC (**Figures 2G,H**), the difference across the six groups was significant [Stimulus Factor, *F*(5,18) = 10.95, *p* < 0.0001]. We found that startle pulses increased by 1.7-fold in the number of c-Fos positive cells in comparison to the control group [*t*(18) = 5.382, *p* = 0.0006, *n* = 4 per group; **Figure 2I**]. Inhibition of the startle by the optimal gaps (-16 dB SPL) and optimal pre-pulses (+12 dB SPL) caused a significant decrease of c-Fos induction, down to control levels [in comparison to startle pulses only – PPI: *t*(18) = 6.338, *p* < 0.0001; GPIAS: *t*(18) = 3.399, *p* = 0.0479; *n* = 4 per group]. The c-Fos expression of the giant nuclei in the PnC was triggered after the startle stimulus while pre-pulses or gaps did not alter their activation, suggesting that they are not involved in the motor output, but instead only responsive to the startle pulse [in comparison to startle pulses only with an average of six cells per ROI – PPI: *t*(18) = 1.705, *p* > 0.99; GPIAS: *t*(18) = 1.418, *p* > 0.99; *n* = 4 per group, **Figure 2J**]. These findings indicate that the induction of c-Fos in the PnC by startle stimuli is effectively prevented by both pre-pulses and gaps.

### Lack of c-FOS Induction in the LGP after GPIAS

**FIGURE 3:**
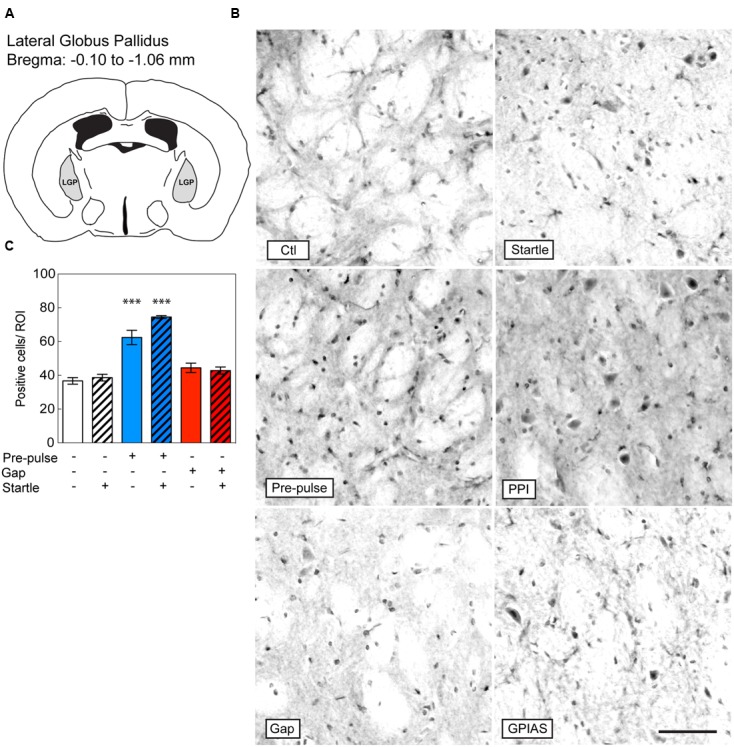
**Induction of c-Fos in the LGP by pre-pulses but not gaps.** Sections were taken from the LGP **(A)**, which is located caudally from the bregma along the rostrocaudal axis between -0.10 and -1.06 μm. **(B)** Representative photomicrographs of c-Fos-immunostaining in the LGP under the six conditions listed in **Figure 2A**. Scale bar, 50 μm. **(C)** Quantification of c-Fos positive cells in the LGP in the six groups. Groups with startle pulses are in hatched bars. Those with pre-pulses are in blue and those with gaps are in red. Data represent means ± SEM (*n* = 4). ^∗∗∗^*P* < 0.0001.

We next investigated the induction of c-Fos in the LGP (**Figure 3A**), known to be involved in the inhibitory pathway of pre-pulse inhibition (Takahashi et al., 2007). In the LGP, the difference across the six groups was significant [Stimulus Factor, *F*(5,18) = 35.44, *p* < 0.0001]. We found that both pre-pulses and PPI increased the number of c-Fos positive cells in the LGP by near twofold in comparison to startle-only groups [pre-pulses: *t*(18) = 9.986, PPI: *t*(18) = 6.624, *p* < 0.0001, *n* = 4 per group; **Figures 3B,C**]. In contrast, gaps or GPIAS did not trigger any additional c-Fos staining when compared to the control or startle-only groups [GAP16: *t*(18) = 1.623, *p* > 0.99; GPIAS: *t*(18) = 1.171, *p* > 0.99; *n* = 4 per group]. These findings strongly suggest that the LGP is not involved in the inhibitory effects caused by gaps on startle suppression.

### GPIAS Triggers c-Fos Expression in the AC unlike PPI

**FIGURE 4:**
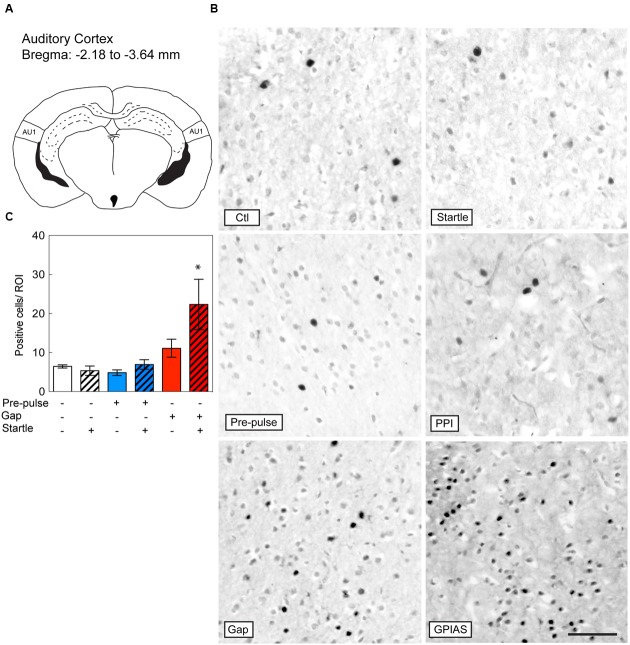
**Induction of c-Fos in the Auditory Cortex by GPIAS only.** Sections were taken from the primary auditory cortex (AC) **(A)**, which is located caudally from the bregma along the rostrocaudal axis between -2.18 and -3.64 mm. **(B)** Representative photomicrographs of c-Fos-immunostaining in the primary AC under the six conditions listed in **Figure 2A**. Scale bar, 50 μm. **(C)** Quantification of c-Fos positive cells in the LGP in the six groups. Groups with startle pulses are in hatched bars. Those with pre-pulses are in blue and those with gaps are in red. Data represent means ± SEM (*n* = 4). ^∗^*P* < 0.05.

Based on studies that have shown the important contribution of the AC in gap detection (Ison et al., 1991; Bowen et al., 2003; Weible et al., 2014), we evaluated the changes in c-Fos positive cells in the primary AC under the six conditions and the difference across the six groups was significant [Stimulus Factor, *F*(5,18) = 5.253, *p* = 0.0038; **Figure 4A**]. With the exception of GPIAS, none of the other conditions triggered c-Fos staining [GPIAS: *t*(18) = 3.864, *p* = 0.0171, *n* = 4 per group; **Figures 4B,C**]. Varying the carrier sound intensities of the control session (e.g., 77, 81 or 100 dB SPL) had no effect on c-Fos activation in the primary AC (data not shown). These results indicate that only the inhibition of the startle pulse by gaps triggers c-Fos induction in the primary AC.

### Baclofen Does Not Rescue from D-amp or MK-801 Disruption of PPI and GPIAS

**FIGURE 5:**
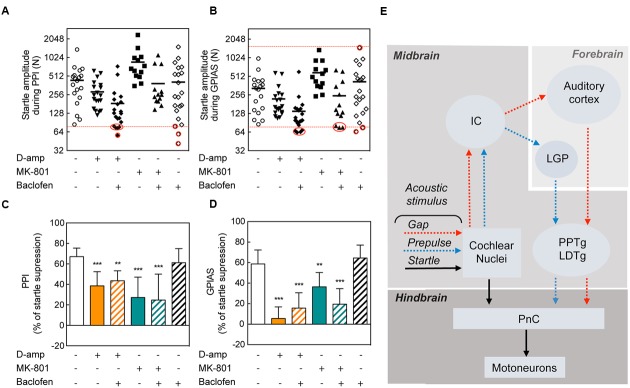
**Lack of rescuing effects of baclofen on PPI and GPIAS.** Startle amplitude during PPI **(A)** and GPIAS **(B)** sessions under different drug conditions. The dots represent each individual’s average startle response during pulse-alone trials for each drug-treatment condition. The dotted red line determines the higher amplitude limit (targeted by the greatest startle amplitude in the drug-administered groups) and/or the lower amplitude limit (targeted by the lowest startle amplitude in the control group). Red circles indicate the excluded animals. Average PPI **(C)** and GPIAS **(D)** scores of the two highest pre-pulse (+6 and +12 dB SPL) and gap (-11 and -16 dB SPL) stimuli. Data represent means ± SEM (*n* = 10–19). ^∗∗^*P* < 0.01, ^∗∗∗^*P* < 0.0001. **(E)** Schematic of the neuronal pathways involved in PPI and GPIAS. The acoustic startle reflex (arrows in black) is composed of three neurons: a neuron in the PnC that transfers acoustic stimulus from the cochlear nucleus to a muscular reaction. Pre-pulses (blue arrows) or gaps (red arrows) are relayed from the cochlear nuclei to the inferior colliculus (IC). In the forebrain, pre-pulses will be directed to the LGP whereas gaps are directed to the AC. Both pre-pulse and gaps will modulate the pedunculopontine nucleus (PPTg), which will inhibit the PnC.

In an attempt to restore the disruption of PPI and GPIAS caused by D-amp and MK-801, we pre-administered baclofen, a GABA_B_ receptor agonist that has been previously shown to block the effects of methamphetamine on PPI (Arai et al., 2008). Since both D-Amp and MK-801 appeared as effective disruptors of PPI and GPIAS (**Figures 1B,C**), we sought to determine whether pharmacological treatment could restore normal PPI and GPIAS. We pre-treated animals with baclofen 15 min prior the administration of D-Amp or MK-801 and performed serial PPI and GPIAS sessions. To exclude the potential influence of differences in startle responses, we performed an exclusion based on a criteria previously described by Stadlbauer et al. (2013). Here, both saline or baclofen groups served as controls. Animals from any of the drug-administered groups for which a startle amplitude was lower than the startle response from control groups were excluded. Animals from the control group for which a startle amplitude was higher than any drug-administered group were excluded as well (**Figures 5A,B**). Analysis of the average pre-pulses and gaps of the greatest differences against the carrier noise (+6 and +12 db SPL in PPI and -11 and -16 dB SPL in GPIAS) stimuli allowed to summarize the differences as a whole and establish that baclofen had no effect on D-amp- or MK-801-induced PPI and GPIAS disruption [PPI MK80-1: *t*(172) = 1.329, *p* > 0.9999; PPI D-Amp: *t*(172) = 1.150, *p* > 0.9999; GPIAS MK80-1: *t*(172) = 2.350, *p* = 0.2986; GPIAS D-Amp: *t*(172) = 1.852, *p* = 0.9854; *n* = 10–18 per group; **Figures 5C,D**].

## Discussion

The salient features of this study reveal that the processing of information during a GPIAS test involves the AC but not the LGP. This indicates that distinct neural nuclei are recruited during GPIAS or PPI to inhibit the ASR. It is interesting to note that a gap within a carrier noise (decreasing intensity) triggers a similar inhibitory response as a pre-pulse (increased intensity) but the two paradigms rely on two different brain regions. This study establishes a framework to better understand the networks involved in GPIAS and therefore improve the understanding of its applicability to temporal processing disorders as well as auditory processing disorders and tinnitus.

We have used c-Fos mapping to compare brain regions involved in GPIAS and PPI. This method has been successfully applied to identify regions involved in PPI (Takahashi et al., 2007; Arai et al., 2008). We found that, in contrast to pre-pulses, gaps embedded in a carrier background do not elicit c-Fos activity in the LGP. This is giving a clear neuroanatomical distinction between these two behavioral paradigms and could help better understand the regulation of temporal information encoded by sounds. Corroborating our findings, it has been found that the LGP is involved in PPI (Takahashi et al., 2007). Electrolytic ablations or local inactivation of the LGP by lidocaine have shown to affect the response to pre-pulses (Takahashi et al., 2007). The involvement of GABA_B_ receptors in the control of PPI has been suggested with the local injection of phaclofen (a GABA_B_ receptors antagonist) in the PPTg. Since GABAergic neurons from the LGP project directly to the PPTg to control the startle response (Takahashi et al., 2007), it has been hypothesized that the LGP controls PPTg function through GABAergic modulation. In contrast to our expectations, baclofen administration, which enhances GABAergic function via GABA_B_ receptors, was not able to restore D-amp- or MK-801-induced PPI or GPIAS disruption. Even though we tested a range of doses (2.5, 5, 7.5, and 10 mg/kg), none were successful in rescuing the phenotype (data not shown). The effects of baclofen are complicated because different studies have shown mixed results in the rescue of PPI (Bortolato et al., 2004; Arai et al., 2008; Frau et al., 2014). This could be due to several reasons including the species or strain used, the analog of amphetamine (e.g., methamphetamine or D-amphetamine), and the dose. For instance, rats have been shown to be responsive to the rescuing effects of baclofen on MK-801-mediated PPI disruption unlike C57BL/6J mice. Nonetheless, our findings suggest that PPI and GPIAS are GABA_B_-independent in the C57 strain.

We found that GPIAS triggered c-Fos activation in the AC. Pioneering work from Ison et al. (1991) has demonstrated in rats the involvement of the AC in GPIAS but not in PPI (Bowen et al., 2003). Temporary and reversible inhibition of cortical activity via the application of high concentrations of potassium chloride (which does not affect the startle response amplitude) disrupted GPIAS but not PPI (Ison et al., 1991). However, when the end of the gap signal is coupled to the onset of the startle stimulus, Ison et al. (1991) were able to reveal that the noise offset itself does not require cortical control. Lesioning of the AC yielded similar conclusions (Bowen et al., 2003). It is possible that gaps presented at different lead times recruit different operating mechanisms. For instance, in rats gap detection at distal lead times (>40 ms) requires muscarinic receptor function, which is not the case at shorter lead times (Ison and Bowen, 2000). Whether this also applies to mice is unknown. Previous work from Ison et al. (1991) identified in rats a biphasic response on startle suppression depending on the interstimulus interval, which is something we did not observe in mice (Yu et al., 2016). In the present study, we used short gaps of 20 ms, presented at 15 ms lead times and showed these could trigger c-Fos staining in the AC in presence of startle stimuli. It is thus possible that the recruitment of cortical function differs between species. Another possibility is that pre-pulse inhibition of the startle reflex could have been sensed by the AC if shorter lead times would have been used. This possibility will require further evaluations in the future.

Studies in rodents have suggested a number of tinnitus neuronal correlates in the AC that translate into (i) an increase in SFR, (ii) neuronal synchrony, (iii) tonotopic reorganization (Elgoyhen et al., 2015; Shore et al., 2016). Such changes could be the underlying cause of the inability of tinnitus-experiencing rodents to have efficient startle suppression by gaps. Weible et al. (2014) identified that comparisons between pre- and post-gap neural activity determines the efficacy in GPIAS. In the AC, parvalbumin-expressing and somatostatin-expressing GABAergic interneurons exert their inhibitory activity on CaMKII-expressing pyramidal neurons to regulate GPIAS (Weible et al., 2014). Interestingly, the optogenetic inactivation of inhibitory interneurons in the AC before or after the gap enhances GPIAS, however, when this is performed throughout pre-, during and post-gap, GPIAS could not be altered (Weible et al., 2014). We thus believe that the continuous pharmacological action of baclofen on the AC throughout the entire trial (pre-, during and post-gap) could not restore GPIAS. This illustrates the limitations of pharmacological approaches in testing the mechanisms of gap detection when compared to the accuracy of optogenetic approaches. We propose an initial model of the regulation of PPI and GPIAS, where differences in the modulation of the inhibitory path occur at the level of the forebrain, in the LGP and the AU, respectively (**Figure 5E**).

A potential application of the present findings to humans would be that, in the event such modulation of AC activity would be detectable during GPIAS for instance by using electroencephalography (EEG) or other non-invasive neuroimaging techniques, such as positron emission tomography (PET), we would predict responses to be altered in presence of tinnitus. As the ASR in humans is highly variable (Braff et al., 2001), and its reliability in presence of tinnitus questioned (Fournier and Hebert, 2013), such measures could become an interesting alternative to blinking responses. Since ABR GIN responses closely reproduce behavioral startle responses in presence of gaps (Lowe and Walton, 2015), a focus on the AC response during gaps in noise appears as a feasible path.

Whereas the role of the AC in GPIAS has been evidenced here, it remains to be determined whether GPIAS has similar or additional nuclei involved in the inhibitory pathway compared to PPI. The neural pathways that control the inhibition of the ASR by pre-pulses is established in higher order regions such as the ventral hippocampus-medial prefrontal cortex (mPFC), as well as the “CSPP” circuitry involving the limbic cortex, striatum, pallidal or pontine tegmentum (Swerdlow et al., 2008, 2016). Acoustic pre-pulses are also regulated via the IC, superior colliculus (SC), pedunculopontine tegmental nucleus (PPTg) and substantia nigra pars compacta (Leitner and Cohen, 1985; Koch et al., 1993; Li et al., 1998; Koch et al., 2000). The large connectivity network involved in PPI emphasizes the need of performing similar studies for GPIAS in order to better understand the factors that modulate the inhibition by gaps and how reliable this paradigm is in the context of assessment of psychiatric and auditory processing disorders as well as tinnitus. The recent findings that tinnitus is involving a large number of non-auditory brain areas (Chen et al., 2015; Sedley et al., 2015) predicts that other brain regions [e.g., amygdala, hippocampus, cerebellum, medial geniculate body, and the reticular formation (Eggermont, 2016)] may alter the efficacy of startle suppression by gaps.

## Conclusion

Gap pre-pulse inhibition of the acoustic startle and PPI both rely on sound cues but utilize different neural pathways to regulate the inhibition of the startle response. These different sound cues, characterized by either an increase (pre-pulses) or a decrease (gaps) in intensity, are recruiting different brain regions. Pre-pulses are activating the LGP to inhibit the startle response, while gaps bypass the LGP to activate the AC. These results are establishing a neuroanatomical foundation for understanding gap detection and its applicability in the context of neurological disorders including auditory processing disorders and tinnitus.

## Ethics Statement

Experimental procedures on animals were performed in accordance with the guidelines and regulations set out by Stockholm’s Norra Djurförsöksetiska Nämnd (N156/14).

## Author Contributions

RM-P carried out the experiments. RM-P, BC, and CC analyzed the results and designed the research. BC and CC directed the research. RM-P, BC, and CC discussed the results and wrote the manuscript. All authors reviewed the manuscript.

## Conflict of Interest Statement

The authors declare that the research was conducted in the absence of any commercial or financial relationships that could be construed as a potential conflict of interest.
