# Supplementary material for: Differential Neural Responses Underlying the Inhibition of the Startle Response by Pre-Pulses or Gaps in Mice
Source: Front Cell Neurosci. 2017 Feb 7;11:19. doi: 10.3389/fncel.2017.00019 (PMC5302757; doi:10.3389/fncel.2017.00019)
Supplement: Supplementary file 1 [file Data_Sheet_1.DOCX]

**Session and trial order for the assessment of drug effects on PPI and GPIAS**

## A) PPI session

Background Analog Level: 412 (corresponding here to 65 dB SPL)

No. of Record Samples: 1000

Samples per Second: 1000

Inter-Trial Interval # 1: 14

Inter-Trial Interval # 2: 10

Inter-Trial Interval # 3: 10

Inter-Trial Interval # 4: 9

Inter-Trial Interval # 5: 15

Inter-Trial Interval # 6: 11

Inter-Trial Interval # 7: 9

Inter-Trial Interval # 8: 14

Inter-Trial Interval # 9: 11

Trial # 1: P114

Trial # 2: P114

Trial # 3: P114

Trial # 4: P114

Trial # 5: P114

Trial # 6: PP6P114

Trial # 7: PP3P114

Trial # 8: nostim

Trial # 9: PP12P114

Trial # 10: P114

Trial # 11: PP3P114

Trial # 12: PP12P114

Trial # 13: P114

Trial # 14: nostim

Trial # 15: PP3P114

Trial # 16: PP6P114

Trial # 17: PP12P114

Trial # 18: nostim

Trial # 19: P114

Trial # 20: PP6P114

Trial # 21: P114

Trial # 22: PP6P114

Trial # 23: PP12P114

Trial # 24: P114

Trial # 25: PP3P114

Trial # 26: nostim

Trial # 27: PP6P114

Trial # 28: PP3P114

Trial # 29: P114

Trial # 30: PP12P114

Trial # 31: PP3P114

Trial # 32: P114

Trial # 33: PP3P114

Trial # 34: PP12P114

Trial # 35: PP6P114

Trial # 36: nostim

Trial # 37: P114

Trial # 38: PP6P114

Trial # 39: PP12P114

Trial # 40: nostim

Trial # 41: P114

Trial # 42: PP3P114

Trial # 43: PP6P114

Trial # 44: P114

Trial # 45: PP6P114

Trial # 46: nostim

Trial # 47: PP12P114

Trial # 48: P114

Trial # 49: PP3P114

Trial # 50: PP12P114

Trial # 51: nostim

Trial # 52: P114

Trial # 53: PP12P114

Trial # 54: PP3P114

Trial # 55: P114

Trial # 56: P114

Trial # 57: P114

Trial # 58: P114

Trial # 59: P114

Sequence Repetitions:1

Acclimation Period (mins):5

***Trial Name: P114***

At 0 Milliseconds: Record Data

At 0 Milliseconds: Analog Level: 725

At 0 Milliseconds: Wait Length (ms): 40

At 40 Milliseconds: Background

At 40 Milliseconds: End of Trial

***Trial Name: PP3P114***

At 0 Milliseconds: Record Data

At 0 Milliseconds: Analog Level: 425

At 0 Milliseconds: Wait Length (ms): 50

At 50 Milliseconds: Background

At 50 Milliseconds: Wait Length (ms): 70

At 120 Milliseconds: Analog Level: 725

At 120 Milliseconds: Wait Length (ms): 20

At 140 Milliseconds: Background

At 140 Milliseconds: End of Trial

***Trial Name: PP6P114***

At 0 Milliseconds: Record Data

At 0 Milliseconds: Analog Level: 450

At 0 Milliseconds: Wait Length (ms): 50

At 50 Milliseconds: Background

At 50 Milliseconds: Wait Length (ms): 70

At 120 Milliseconds: Analog Level: 725

At 120 Milliseconds: Wait Length (ms): 20

At 140 Milliseconds: Background

At 140 Milliseconds: End of Trial

***Trial Name: PP12P114***

At 0 Milliseconds: Record Data

At 0 Milliseconds: Analog Level: 485

At 0 Milliseconds: Wait Length (ms): 50

At 50 Milliseconds: Background

At 50 Milliseconds: Wait Length (ms): 70

At 120 Milliseconds: Analog Level: 725

At 120 Milliseconds: Wait Length (ms): 20

At 140 Milliseconds: Background

At 140 Milliseconds: End of Trial

***Trial Name: nostim***

At 0 Milliseconds: Record Data

At 0 Milliseconds: Background

At 0 Milliseconds: Wait Length (ms): 10

At 10 Milliseconds: End of Trial

## B) GPIAS session

As the system from San Diego Instruments does not allow to generate gaps into a continuous background carrier, we generated trials with a long initial time of varying duration and intensities in which a gap of a level down to floor level (background) was generated.

Background Analog Level: 412

No. of Record Samples: 1000

Samples per Second: 1000

Inter-Trial Interval # 1: 1

Trial # 1: 65P114

Trial # 2: 65P114

Trial # 3: 65P114

Trial # 4: 65P114

Trial # 5: 65P114

Trial # 6: GAP11P114t8

Trial # 7: GAP6P114t9

Trial # 8: GAP81nostim

Trial # 9: GAP16P114t12

Trial # 10: 65P114

Trial # 11: GAP6P114t13

Trial # 12: GAP16P114t14

Trial # 13: 65P114

Trial # 14: GAP81nostim

Trial # 15: GAP6P114t10

Trial # 16: GAP11P114t9

Trial # 17: GAP16P114t8

Trial # 18: GAP71nostim

Trial # 19: 65P114

Trial # 20: GAP11P114t10

Trial # 21: 65P114

Trial # 22: GAP11P114t15

Trial # 23: GAP16P114t15

Trial # 24: 65P114

Trial # 25: GAP6P114t9

Trial # 26: GAP77nostim

Trial # 27: GAP11P114t11

Trial # 28: GAP6P114t8

Trial # 29: 65P114

Trial # 30: GAP16P114t9

Trial # 31: GAP6P114t11

Trial # 32: 65P114

Trial # 33: GAP6P114t12

Trial # 34: GAP16P114t12

Trial # 35: GAP11P114t12

Trial # 36: GAP71nostim

Trial # 37: 65P114

Trial # 38: GAP11P114t13

Trial # 39: GAP16P114t11

Trial # 40: GAP71nostim

Trial # 41: 65P114

Trial # 42: GAP6P114t14

Trial # 43: GAP11P114t14

Trial # 44: 65P114

Trial # 45: GAP11P114t8

Trial # 46: GAP81nostim

Trial # 47: GAP16P114t13

Trial # 48: 65P114

Trial # 49: GAP6P114t8

Trial # 50: GAP16P114t15

Trial # 51: GAP77nostim

Trial # 52: 65P114

Trial # 53: GAP16P114t9

Trial # 54: GAP6P114t15

Trial # 55: 65P114

Trial # 56: 65P114

Trial # 57: 65P114

Trial # 58: 65P114

Trial # 59: 65P114

Sequence Repetitions:1

Acclimation Period (mins):5

***Trial Name: 65P114***

At 0 Milliseconds: Background

At 0 Milliseconds: Wait Length (ms): 9999

At 9999 Milliseconds: Wait Length (ms): 2000

At 11999 Milliseconds: Record Data

At 11999 Milliseconds: Analog Level: 725

At 11999 Milliseconds: Wait Length (ms): 20

At 12019 Milliseconds: Background

At 12019 Milliseconds: Wait Length (ms): 500

At 12519 Milliseconds: End of Trial

**Trial Name: GAP6P114t15**

At 0 Milliseconds: Analog Level: 450

At 0 Milliseconds: Wait Length (ms): 9999

At 9999 Milliseconds: Wait Length (ms): 5000

Total duration of the first analog level corresponds here to 15 seconds. 7 trials should be created with analog durations varying with 1 second from 15 seconds down to 8 seconds.

At 14999 Milliseconds: Record Data

At 14999 Milliseconds: Background

At 14999 Milliseconds: Wait Length (ms): 50

At 15049 Milliseconds: Analog Level: 450

At 15049 Milliseconds: Wait Length (ms): 15

At 15064 Milliseconds: Analog Level: 725

At 15064 Milliseconds: Wait Length (ms): 20

At 15084 Milliseconds: Analog Level: 450

At 15084 Milliseconds: Wait Length (ms): 1000

At 16084 Milliseconds: End of Trial

**Trial Name: GAP11P114t15**

At 0 Milliseconds: Analog Level: 475

At 0 Milliseconds: Wait Length (ms): 9999

At 9999 Milliseconds: Wait Length (ms): 5000

At 14999 Milliseconds: Record Data

At 14999 Milliseconds: Background

At 14999 Milliseconds: Wait Length (ms): 50

At 15049 Milliseconds: Analog Level: 475

At 15049 Milliseconds: Wait Length (ms): 15

At 15064 Milliseconds: Analog Level: 725

At 15064 Milliseconds: Wait Length (ms): 20

At 15084 Milliseconds: Analog Level: 475

At 15084 Milliseconds: Wait Length (ms): 1000

At 16084 Milliseconds: End of Trial

**Trial Name: GAP16P114t15**

At 0 Milliseconds: Analog Level: 512

At 0 Milliseconds: Wait Length (ms): 9999

At 9999 Milliseconds: Wait Length (ms): 5000

At 14999 Milliseconds: Record Data

At 14999 Milliseconds: Background

At 14999 Milliseconds: Wait Length (ms): 50

At 15049 Milliseconds: Analog Level: 512

At 15049 Milliseconds: Wait Length (ms): 15

At 15064 Milliseconds: Analog Level: 725

At 15064 Milliseconds: Wait Length (ms): 20

At 15084 Milliseconds: Analog Level: 512

At 15084 Milliseconds: Wait Length (ms): 1000

At 16084 Milliseconds: End of Trial

**Trial Name: GAP71nost**im

At 0 Milliseconds: Record Data

At 0 Milliseconds: Analog Level: 450

At 0 Milliseconds: Wait Length (ms): 9999

At 9999 Milliseconds: Wait Length (ms): 5000

At 14999 Milliseconds: End of Trial

**Trial Name: GAP77nostim**

At 0 Milliseconds: Analog Level: 485

At 0 Milliseconds: Record Data

At 0 Milliseconds: Wait Length (ms): 9999

At 9999 Milliseconds: Wait Length (ms): 5000

At 14999 Milliseconds: End of Trial

**Trial Name: GAP81nostim**

At 0 Milliseconds: Record Data

At 0 Milliseconds: Analog Level: 512

At 0 Milliseconds: Wait Length (ms): 9999

At 9999 Milliseconds: Wait Length (ms): 5000

At 14999 Milliseconds: End of Trial
