# Supplementary material for: Differential Neural Responses Underlying the Inhibition of the Startle Response by Pre-Pulses or Gaps in Mice
Source: Front Cell Neurosci. 2017 Feb 7;11:19. doi: 10.3389/fncel.2017.00019 (PMC5302757; doi:10.3389/fncel.2017.00019)
Supplement: Supplementary file 2 [file Data_Sheet_2.DOCX]

**Trials for the assessment of c-Fos induction in the brain**

## A) Control trials

**Trial Name: Background**

At 0 Milliseconds: Analog Level: 478

At 0 Milliseconds: Record Data

At 0 Milliseconds: Wait Length (ms): 9999

At 9999 Milliseconds: End of Trial

## B) Startle only trials

***Trial Name: P114***

At 0 Milliseconds: Record Data

At 0 Milliseconds: Analog Level: 725

At 0 Milliseconds: Wait Length (ms): 40

At 40 Milliseconds: Background

At 40 Milliseconds: End of Trial

## C) Pre-pulse only trials

**Trial Name: PP10**

At 0 Milliseconds: Record Data

At 0 Milliseconds: Analog Level: 478

At 0 Milliseconds: Wait Length (ms): 50

At 50 Milliseconds: Background

At 50 Milliseconds: Wait Length (ms): 90

At 140 Milliseconds: End of Trial

## D) Pre-pulse and pulse

**Trial Name: PP10P114**

At 0 Milliseconds: Record Data

At 0 Milliseconds: Analog Level: 478

At 0 Milliseconds: Wait Length (ms): 50

At 50 Milliseconds: Background

At 50 Milliseconds: Wait Length (ms): 70

At 120 Milliseconds: Analog Level: 725

At 120 Milliseconds: Wait Length (ms): 20

At 140 Milliseconds: Background

At 140 Milliseconds: End of Trial

## E) Gap only trials

**Trial Name: GAP16**

At 0 Milliseconds: Analog Level: 512

At 0 Milliseconds: Wait Length (ms): 9999

At 9999 Milliseconds: Record Data

At 9999 Milliseconds: Background

At 9999 Milliseconds: Wait Length (ms): 50

At 10049 Milliseconds: Analog Level: 512

At 10049 Milliseconds: Wait Length (ms): 1000

At 11049 Milliseconds: End of Trial

## F) Gap and startle trials

**Trial Name: GAP16P114t15**

At 0 Milliseconds: Analog Level: 512

At 0 Milliseconds: Wait Length (ms): 9999

At 9999 Milliseconds: Wait Length (ms): 5000

At 14999 Milliseconds: Record Data

At 14999 Milliseconds: Background

At 14999 Milliseconds: Wait Length (ms): 50

At 15049 Milliseconds: Analog Level: 512

At 15049 Milliseconds: Wait Length (ms): 15

At 15064 Milliseconds: Analog Level: 725

At 15064 Milliseconds: Wait Length (ms): 20

At 15084 Milliseconds: Analog Level: 512

At 15084 Milliseconds: Wait Length (ms): 1000

At 16084 Milliseconds: End of Trial
